# Cytokine signaling as key regulator of pathological angiogenesis in the eye

**DOI:** 10.1016/j.ebiom.2021.103662

**Published:** 2021-10-28

**Authors:** Thomas Langmann

**Affiliations:** a Laboratory for Experimental Immunology of the Eye, Department of Ophthalmology, University of Cologne, Faculty of Medicine and University Hospital Cologne, D-50931 Cologne, Germany; b Center for Molecular Medicine Cologne (CMMC), University of Cologne, D-50931 Cologne, Germany

Pathological events leading to uncontrolled formation of nascent, leaky blood vessels and their penetration through the inner and outer retina are hallmarks of diabetic retinopathy and “wet” age-related macular degeneration (AMD), respectively [1]. The prevalence of these two retinal neovascular diseases is constantly increasing in the ageing population and they are considered as leading causes for severe visual impairment and even blindness [2].

Nearly 30 years of research have discovered that ocular dysregulation of angiogenic growth factors including vascular endothelial growth factors (VEGF) and placental growth factor (PGF) trigger and sustain the formation of abnormal leaky blood vessels in the eye [3], [4], [5]. Since these two proteins and other members of the VEGF growth factor family are mainly active as secreted molecules, the cellular source in the diseased retina is often unclear. In addition to their direct angiogenic effects on endothelial cells, VEGF-A and PGF can elicit potent inflammatory signals when bound to their cognate receptor, VEGFR1, on immune cells [5].

The current treatment options for these disease entities exclusively rely on intravitreal injections of VEGF/PGF antibody inhibitors or VEGFR1/R2 chimeric trap molecules. However, these therapies have significant limitations including the burden of frequent intravitreal injections and resistance to continuous and long-term treatment.

Aiming at understanding further key mechanisms of retinal neovascularization and searching for novel treatment targets, and recently published in *EBioMedicine*, Wang *et al.* elegantly studied the role of retinal microinflammation and particularly the contribution of myeloid cells in pathological vessel formation [6]. They used the experimental rodent model of laser-induced choroidal neovascularization, which is a well-established system mimicking vessel leakage of human wet AMD. This model has been previously applied to document the role of long-lived resident retinal microglia that secrete pro-inflammatory cytokines and angiogenic growth factors, and thereby substantially contribute to the formation of retinal neovessels [7]. The study discussed here focuses on the role of infiltrating cells of the myeloid lineage and their signaling via suppressor of cytokine signaling 3 (SOCS3), a negative feedback regulator of ocular inflammation and angiogenesis [8].

A previous study had already indicated that infiltration of myeloid cells, mainly monocytes with increased activity of signal transducer and activator of transcription 3 (STAT3), seem to be critical for neovascular AMD and that genetic deletion of its negative regulator, SOCS3, accelerated choroidal neovascularization (CNV) in mice [9]. In the report presented here, fate mapping experiments using GFP bone marrow chimeric mice and myeloid-specific LysM Cre-driven reporter mice corroborated these earlier data and additionally showed that SOCS3 expression is highly induced in myeloid cells recruited to the CNV lesion area. Myeloid-specific SOCS3 loss of function and complementary gain of function experiments then demonstrated a strong effect on monocyte/macrophage and microglia recruitment, inflammatory gene expression, and pathological CNV formation *in vivo*. Co-culture experiments combining medium collected from SOCS3 knockout or overexpressing macrophages with wild type choroidal explants also showed that myeloid SOCS3 directly affects choroid sprouting *ex vivo*. Finally, in an elegant translational therapy approach, the authors then orally applied the flavonoid naringenin, a SOCS3 gene transcription activator, to lasered mice and found a remarkable 40% suppression of CNV formation after treatment. Furthermore, systemic administration of a 24 amino acid peptide that mimics SOCS3 function showed similar beneficial effects on CNV, albeit with less overall reduction in lesion area.

The paper discussed here clearly highlights the importance of SOCS3 signaling in myeloid cells as a gate keeper of inflammatory cell influx in the damaged retina, suggesting a novel therapeutic approach that is independent from anti-VEGF trapping. Nevertheless, there are several open questions that need to be addressed in future work. First, it will be important to clarify whether the presented SOCS3/STAT3 pathway is also relevant to other diseases that affect abnormal blood vessels in the eye, including diabetic retinopathy. Second, recently performed single cell transcriptomics in the laser-CNV mouse retina identified a complex interaction network of damage-associated resident microglia, recruited monocytes, and other immune cells [10], and it will therefore be important to dissect the role of SOCS3 in these individual cell types. Third, the concept of SOCS3 targeting to treat ocular neovascular diseases is promising but needs a comprehensive analysis of immunological side effects, mode of application (ie, systemic versus intraocular), as well as a thorough evaluation of pharmacological properties of small molecules or peptide mimetics that usually have a short half-life in the ocular compartments.

## Contributors

This commentary was written solely by TL.

## Declaration of Competing Interest

The author declares no conflict of interest
